# Adjuvant Rituximab—Exploratory Trial in Young People With Graves Disease

**DOI:** 10.1210/clinem/dgab763

**Published:** 2021-10-23

**Authors:** Tim D Cheetham, Michael Cole, Mario Abinun, Amit Allahabadia, Tim Barratt, Justin H Davies, Paul Dimitri, Amanda Drake, Zainaba Mohamed, Robert D Murray, Caroline A Steele, Nicola Zammitt, Sonya Carnell, Jonathan Prichard, Gillian Watson, Sophie Hambleton, John N S Matthews, Simon H S Pearce

**Affiliations:** 1 Translational and Clinical Research Institute, Faculty of Medical Sciences, Newcastle University, International Centre for Life, Newcastle upon Tyne, NE1 3BZ, UK; 2 Department of Paediatric Endocrinology, Great North Children’s Hospital, Newcastle upon Tyne Hospitals NHS Foundation Trust, NE1 4LP, UK; 3 Population Health Sciences Institute, Newcastle University, Newcastle upon Tyne, NE2 4AX, UK; 4 Immunity & Inflammation Theme, Translational and Clinical Research Institute, Faculty of Medical Sciences, Newcastle University, Newcastle upon Tyne, NE2 4HH, UK; 5 Department of Paediatric Immunology, Great North Children’s Hospital, Newcastle upon Tyne Hospitals NHS Foundation Trust, Newcastle upon Tyne, NE1 4LP, UK; 6 Academic Directorate of Diabetes and Endocrinology, Royal Hallamshire Hospital, Sheffield, S10 2JF, UK; 7 University of Birmingham, Diabetes Unit, Birmingham Children’s Hospital, Birmingham, B4 6NH, UK; 8 Birmingham Children’s Hospital, Birmingham, B46NH, UK; 9 Department of Paediatric Endocrinology, Faculty of Medicine, University of Southampton, University Hospital Southampton NHS Foundation Trust, Southampton, SO16 6YD, UK; 10 The Department of Paediatric Endocrinology, Sheffield Children’s NHS Trust, Western Bank, Sheffield, S10 2TH, UK; 11 Centre for Cardiovascular Science, Queen’s Medical Research Institute, Edinburgh, EH16 4TJ, UK; 12 Leeds Centre for Diabetes and Endocrinology, Leeds Teaching Hospitals NHS Trust, Leeds, LS97TF, UK; 13 Children and Adolescent services, Leeds Teaching Hospitals NHS Trust, UK; 14 Edinburgh Centre for Endocrinology & Diabetes, Royal Infirmary of Edinburgh, Edinburgh, EH16 4SA, UK; 15 Newcastle Clinical Trials Unit, Newcastle University, Newcastle upon Tyne, NE2 4AE, UK; 16 School of Mathematics, Statistics & Physics, Newcastle University, Newcastle upon Tyne, NE1 7RU, UK; 17 Department of Endocrinology, Royal Victoria Infirmary, Newcastle upon Tyne, NE1 4LP, UK

**Keywords:** thyrotoxicosis, Graves disease, thionamide, rituximab, adolescents, children

## Abstract

**Context:**

Remission rates in young people with Graves hyperthyroidism are less than 25% after 2 years of thionamide antithyroid drug (ATD).

**Objective:**

We explored whether rituximab (RTX), a B-lymphocyte–depleting agent, would increase remission rates when administered with a short course of ATD.

**Methods:**

This was an open-label, multicenter, single-arm, phase 2 trial in young people (ages, 12-20 years) with Graves hyperthyroidism. An A’Hern design was used to distinguish an encouraging remission rate (40%) from an unacceptable rate (20%). Participants presenting with Graves hyperthyroidism received 500 mg RTX and 12 months of ATD titrated according to thyroid function. ATDs were stopped after 12 months and primary outcome assessed at 24 months. Participants had relapsed at 24 months if thyrotropin was suppressed and free 3,5,3′-triiodothyronine was raised; they had received ATD between months 12 and 24; or they had thyroid surgery/radioiodine.

**Results:**

A total of 27 participants were recruited and completed the trial with no serious side effects linked to treatment. Daily carbimazole dose at 12 months was less than 5 mg in 21 of 27 participants. Thirteen of 27 participants were in remission at 24 months (48%, 90% one-sided CI, 35%-100%); this exceeded the critical value (9) for the A’Hern design and provided evidence of a promising remission rate. B-lymphocyte count at 28 weeks, expressed as a percentage of baseline, was related to likelihood of remission.

**Conclusion:**

Adjuvant RTX, administered with a 12-month course of ATD, may increase the likelihood of remission in young people with Graves hyperthyroidism. A randomized trial of adjuvant RTX in young people with Graves hyperthyroidism is warranted.

Graves hyperthyroidism (Graves disease, GD) is a challenging condition for the young person and their family. A low remission rate of 20% to 30% following standard antithyroid drug (ATD) treatment of 1 to 3 years’ duration means that clinicians increasingly recommend treatment for an extended time (1-3). For the majority of patients who still relapse after ATD, the choice is to return to ATD therapy or to opt for definitive treatment with surgery (total thyroidectomy) or radioiodine (RI). A requirement for long-term thyroxine (T4) replacement in these patients is not ideal because the psychological well-being of adults with normal thyrotropin (TSH) concentrations on thyroid hormone replacement is reduced in comparison to controls (4), and quality of life is reduced in patients receiving either RI or thyroidectomy for GD (5).

While many adult patients with GD remit following ATD therapy, some also remit spontaneously (6-8), which suggests a degree of immune system plasticity that can potentially be exploited by modern immunomodulatory agents (9). These agents could ameliorate or “switch off” the pathogenic immune response and increase the likelihood of remission.

Rituximab (RTX) is a chimeric anti-CD 20 monoclonal antibody, first developed for the treatment of B-cell malignancy but later repurposed for immunotherapy, which suppresses humoral immunity by depleting circulating B cells, typically for around 6 months after 1 or 2 doses. RTX is a logical choice of immune therapy in GD because it targets the cells responsible for producing the pathogenic stimulatory antibodies (thyroid receptor antibodies or TRAb). RTX has been used in a range of autoimmune disorders in children and has a favorable safety profile (10-12). Furthermore, RTX has shown some promise in the context of adults with GD and Graves ophthalmopathy (13-17).

We wanted to explore whether immunotherapy with RTX in young patients with GD might increase the likelihood of disease remission when used as an adjunct to a short course of standard ATD.

## Materials and Methods

### Trial Objectives

The primary objective of this trial was to establish whether a single 500-mg dose of RTX, when administered together with a 12-month course of thionamide ATD (carbimazole [CBZ] or propylthiouracil [PTU]) would result in a meaningful improvement in the proportion of young people with Graves hyperthyroidism with prolonged disease remission.

Secondary objectives were to examine the safety of the trial treatment regimen by determining the nature and frequency of adverse events (AEs) and to examine the relationship between remission/relapse status at 24 months and the following parameters:

1. TRAb titer at the time of RTX administration
2. immune cellular response, in particular the B-cell number (CD19 + cells) at 28 and 52 weeks expressed as a percentage of baseline values
3. cumulative dose of ATD
4. the time taken for free T4 (FT4) and free 3,5,3′-triiodothyronine (FT3) to normalize post-RTX administration

### Design

This was an investigator-initiated, open-label, single-arm, single-stage, phase 2 trial using an A’Hern design (18). The trial was funded by the Medical Research Council (MR/N006607/1) and registered with an international standard randomized controlled trial number of 20381716. Details of the trial protocol have been published (19).

At the outset we felt there was insufficient justification for a randomized trial, which would necessitate a much larger number of participants, without first demonstrating an efficacy signal in the young. The approach taken uses predetermined thresholds to decide whether the proposed treatment regimen is likely to meet a minimum level of efficacy before comparing it to standard treatment as part of a randomized trial. The design was constructed to be able to distinguish a promising remission rate of at least 40% from an unacceptable rate of less than 20% with 80% power and a type I error of 10%. A remission rate of more than 40% has not been reported in young people with Graves hyperthyroidism following ATD therapy of 1 to 2 years’ duration. The recruitment target was 27 participants assuming that 10% would be lost to follow-up; this is the smallest number that satisfies the design criteria. A total of 27 patients were recruited and because there were no participants lost to follow-up, the type I error was 7.4% and the power was 81.6%.

### Participants

UK-based patients diagnosed with GD aged between 12 and 20 years inclusive were recruited between October 2016 and August 2018. Eight pediatric endocrine units and 7 adult endocrine centers based in Birmingham, Doncaster, Edinburgh, Newcastle, Leeds, Sheffield, Cardiff, and Southampton were opened to recruitment.

**Figure 1.**
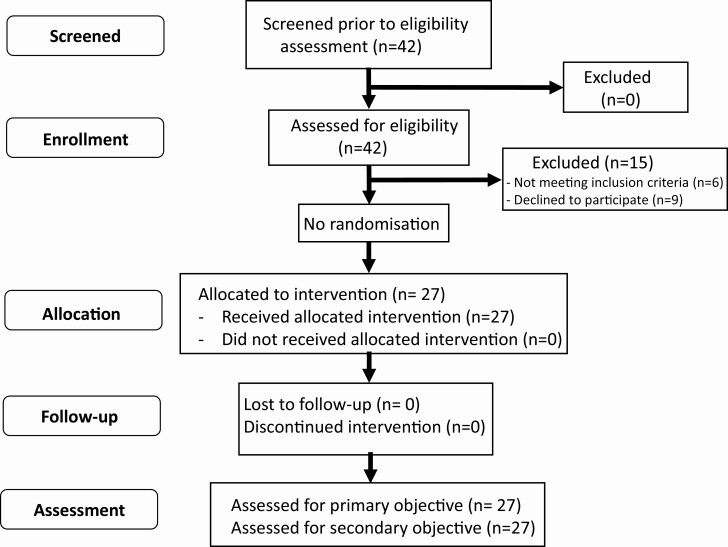
CONSORT diagram.

Participants assented or consented to take part in the trial with additional parental consent in those individuals younger than 16 years. Participants were diagnosed with GD by the clinician on the basis of a clinical and biochemical picture that included a suppressed serum TSH at diagnosis (levels that were unrecordable and hence below the assay threshold according to the local reference range) and raised serum free thyroid hormone concentrations (above the local reference range) in addition to an elevated TRAb. Participants were less than 6 weeks from the initiation of antithyroid drug treatment (CBZ or PTU) for the first time and had not had previous episodes of autoimmune thyroid disease. Patients with significant cardiorespiratory disease, renal, or hepatic disease including hepatitis B and C were excluded. A comprehensive list of inclusion/exclusion criteria are shown in Table 1. The CONSORT diagram is shown in Fig. 1.

**Table 1.** Inclusion and exclusion criteria

Inclusion criteria
• Excess thyroid hormone concentrations at diagnosis: elevated FT3 and/ or free thyroxine (based on local assay)
• Suppressed (unrecordable) TSH (based on local assay)
• Patients ages 12-20 y inclusive who are < 6 wk from initiation of antithyroid drug treatment (CBZ or PTU) for first time
• Elevated thyroid binding-inhibitory immunoglobulin or thyroid receptor antibodies (TRAb including TBII) based on local assay. Patients may or may not have raised TPO antibody titer
• All patients must be willing to use effective forms of contraception for 12 mo posttreatment with RTX
• Female participants of childbearing potential must have a negative pregnancy test at screening. This will need to be repeated the day of RTX administration if more than 7 d has elapsed since screening visit or negative pregnancy test
• Able and willing to adhere to 2-y study period
Exclusion criteria
• Previous episodes of autoimmune thyroid disease
• Patients with active, severe infection (eg, tuberculosis, sepsis, and opportunistic infections) or severely immunocompromised patients
• Patients with known allergy or contraindication to CBZ and PTU
• Participants with previous use of immunosuppressive or cytotoxic drugs (including RTX and methylprednisolone but excluding inhaled glucocorticoid and oral glucocorticoid for asthma or topical glucocorticoid for eczema)
• Chromosomal disorders known to be associated with an increased risk of autoimmune thyroid disease, including Down syndrome and Turner syndrome
• Pregnancy, planned pregnancy during study period, or current breastfeeding
• Absence of informed consent from parent/legal guardian for participants age < 16 y
• Participants with significant chronic cardiac, respiratory, or renal disorder or nonautoimmune liver disease
• Participants with known allergy or contraindication to RTX or methylprednisolone
• Participants with evidence of hepatitis B/C infection, assessed by determining HBsAg status, HB Core antibody status, and HCV antibody status
• Participants in families who know they will be moving out of catchment areas during 2 y following RTX treatment
• Participants currently involved in any other clinical trial of an IMP or who have taken an IMP within 30 d before trial entry

Abbreviations: CBZ, carbimazole; FT3, free 3,5,3′-triiodothyronine; HB Core, hepatitis B core; HBsAg, hepatitis B surface antigen; HCV, hepatitis C virus; IMP, investigational medicinal product; PTU, propylthiouracil; RTX, rituximab; TPO, thyroid peroxidase; TRAb, thyroid receptor antibodies; TSH, thyrotropin.

### Procedures

#### Goiter size

Goiter size was assessed clinically at the time of recruitment as being neither visible nor palpable, palpable but not visible, palpable and visible or large to a point that it was easily seen from 3 or more feet (> 1 m) away.

#### Antithyroid drug therapy

**Figure 2.**
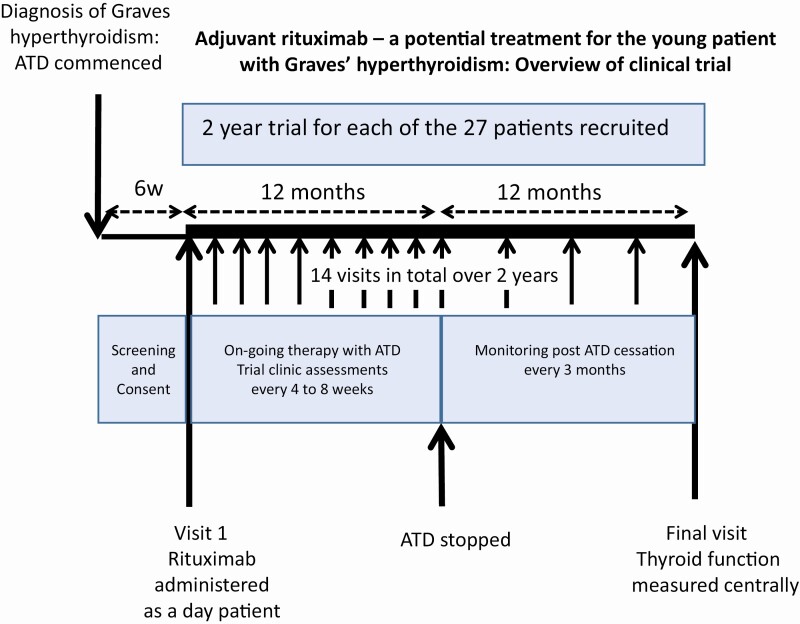
Outline of patient schedule.

The visit schedule is outlined in Fig. 2.

Participants were commenced on thionamide (ATD) with the dose titrated against prevailing thyroid function tests. A dose titration strategy was recommended to reduce the likelihood of AEs that might necessitate early definitive treatment (1). Managing clinicians were experienced endocrinologists familiar with GD management, and a framework suggesting alterations in ATD dose according to prevailing biochemistry was provided within the protocol (19). PTU was commenced as a replacement ATD in the case of AEs that did not involve hepatic dysfunction or neutropenia. ATD treatment was stopped at 12 months with outcomes assessed at 24 months. If patients were thyrotoxic at 12 months then the ATD was continued.

#### Rituximab

Before administering RTX, we established that participants were hepatitis B/C antibody negative and that a pregnancy test was negative in the case of female participants.

A single dose of 500 mg of RTX (MabThera) was administered as an intravenous infusion within 6 weeks of diagnosis. Glucocorticoid (methylprednisolone 125 mg intravenously), antihistamine (chlorpheniramine 10 mg intravenously), and paracetamol (500 mg 12-16 years, 1 g > 16 years) preceded each infusion according to the manufacturer’s recommendation (summary of product characteristics) (20).

#### Schedule and samples

TRAb were measured at primary hospital locations before randomization to confirm a diagnosis of Graves hyperthyroidism. TRAb and thyroid peroxidase antibodies (ATPO) were also measured centrally the day of the RTX infusion (baseline), and at week 52 and week 104.

Participants were reviewed at week 4 post-RTX then at weeks 8, 12, 16, 20, 28, 36, 44, 52, 65, 78, 91, and 104 (see Fig. 2). Thyroid function tests (TSH, FT4, and FT3) were taken at each visit.

Additional samples taken intermittently included a full blood count (hemoglobin, platelets, and white cells—neutrophils and lymphocytes) at baseline and weeks 4, 12, 28, 36, 52, and 104 and, if participants were on PTU, liver function tests (bilirubin, alanine transaminase) at each visit. We also measured immunological indices including B cells (CD 19) at baseline and weeks 4, 12, 28, 36, 52, and 104; serum immunoglobulin levels (IgG, IgM, IgA) at baseline and weeks 12, 28, 36, 52 and 104 and specific antibody titers to tetanus, *Haemophilus influenza* group b (Hib) and Pneumococcus at baseline, weeks 52 and 104.

### Participant Safety

AEs were graded as mild, moderate, or severe and relationship to trial medication as unrelated, unlikely, possible, probable, or definite. For serious adverse reactions, an assessment of expectedness was performed.

All events were coded systematically at the end of the trial using the Medical Dictionary for Regulatory Activities (MedDRA) version 22.0. The Clinical Data Management System auto-coded the majority of AEs to the latest version of the MedDRA dictionary. If this was not possible, AEs were coded from the MedDRA dictionary by the chief investigator (T.D.C.).

### Central Analyses

TSH was measured using the Roche Elecsys TSH assay (Roche Diagnostics GmbH). The lower and upper reporting limits are 0.05 and 1000 mIU/L. Interassay precision is 5.7% at 0.1 mIU/L and 2.3% at 29.7 mIU/L. FT4 was measured using the Roche Elecsys FT4 III assay. The lower and upper reporting limits are 1.3 and 100 pmol/L. Interassay precision is 4.3% at 10.5 pmol/L and 3.9% at 36.4 pmol/L. FT3 was measured using the Roche Elecsys FT3 III assay. The lower and upper reporting limits are 1.5 and 50 pmol/L. Interassay precision is 3.3% at 5.0 pmol/L and 2.3% at 23.2 pmol/L. ATPO were measured using the Roche Elecsys Anti-TPO assay. The lower and upper reporting limits are 5 and 600 kU/L. Interassay precision is 10.8% at 37.4 kU/L and 5.9% at 79.3 kU/L. TSH receptor antibodies were measured using the BRAHMS TRAK human Kryptor assay (Brahms GmbH). The lower and upper reporting limits are 1 and 100 IU/L. Interassay precision is 9.2% at 2.7 IU/L and 4.3% at 9.9 IU/L.

Pneumococcal, tetanus, and Hib antibodies were measured using an antipneumococcal capsular polysaccharide IgG enzyme immunoassay kit, an antitetanus toxoid IgG enzyme immunoassay kit, and an anti–Hib enzyme immunoassay kit (VaccZyme) with an analytical sensitivity of 0.62 mg/L, 0.01 to 7 IU/mL, and 0.11 mg/L, respectively.

Lymphocyte subsets were performed using BD TruCount technology (Becton, Dickinson). A total of 50 µL of EDTA anticoagulated peripheral blood is incubated with BD Multitest 4-color TBNK reagent for 15 minutes in the dark. Then, 2 mL of fluorescence-activated cell sorter (FACS) Lyse is added for red blood cell lysis. Following a further 15-minute incubation, the sample is ready for collection on the flow cytometer (BD FACS Canto II). For patients on RTX for whom the CD19+ B-cell count is calculated to be less than 1% of lymphocytes, samples are reacquired to collect 40 000 lymphocytes in total. This ensures sufficient cells have been assessed to be confident in reporting very low numbers, particularly an absolute count of 0 cells/µL.

### Statistical Analysis

The primary end point of the trial was the number of participants in remission at 2 years following a single dose of RTX and a 12-month course of ATD (the same as the number of participants who had not relapsed). Participants were deemed to have relapsed if they received any concomitant ATD in the trial 52 weeks or more post-RTX administration (± 14 days), or had undergone surgical (thyroidectomy) or RI treatment because of hyperthyroidism at any time following RTX administration. They were also considered to have relapsed if serum TSH was less than the lower limit of the normal laboratory reference range and serum FT3 was above the upper limit of the normal laboratory reference range at 2 years (central biochemical analysis in the Newcastle laboratory for TSH and FT3).

The number of participants in remission 2 years after RTX administration was compared to the appropriate A’Hern critical number (18); this number was prespecified as 9 for the sample size and design parameters of this trial. The remission rate is reported with a one-sided 90% CI calculated using exact methods.

The distributions of the secondary outcome measures were compared between participants deemed in and out of remission at 24 months by Mann-Whitney test, with bootstrap CIs for the difference in medians. Analysis of cumulative ATD dose replaces PTU dose with a CBZ “equivalent dose” assumed to be a tenth of the PTU dose.

## Results

### Participant Baseline Characteristics

Twenty seven participants (24 female) were recruited from 5 pediatric endocrine centers (Birmingham, Leeds, Newcastle, Sheffield, Southampton) and 4 adult endocrine centers (Edinburgh, Leeds, Newcastle, Sheffield), and all 27 completed the trial. The mean patient age at recruitment was 15.3 years with a range of 12.2 years to 20.9 years. Goiter size was assessed in 26 patients at the time of recruitment and identified as being neither visible nor palpable in 6 participants, palpable but not visible in 10 participants, palpable and visible in 8 participants, and large to a point that it was easily seen from 3 or more feet (> 1 m) away in 2 participants. The baseline characteristics of participants are summarized in Table 2.

**Table 2.** Participant baseline characteristics

Variable	Mean	SD	Median	Minimum	Maximum	No.
Age, y (24 female, 3 male)	15.3	2.39	15.0	12.2	20.9	27
Height, cm	162.9	6.86	162.7	149.0	180.6	27
Weight, kg	58.2	15.99	55.2	31.2	121.9	27
BMI	21.7	4.26	21.1	14.1	37.4	27
BMI SD score	0.43	1.19	0.56	–2.80	3.33	27
TSH, mU/L	0.08	0.23	0.05	0.01	1.21	27
FT3, pmol/L	10.85	7.12	8.05	2.5	29.6	22
FT4, pmol/L	23.66	14.46	18.5	5.2	57.2	27
Thyroid antibodies: TPO, kU/L	326.2	450.4	162	3	2000	25
Thyroid antibodies: TRAb, U/L	14.6	14.59	8.7	1.2	50.7	22

Participants may have been taking antithyroid drugs for up to 6 weeks before baseline visit. Reference ranges based on central laboratory: TSH ages 12 to 18 years, 0.5 to 4.3 mU/L; TSH older than 18 years, 0.3 to 4.5 mU/L; FT3 ages 12 to 18 years, 3.9 to 7.7 pmol/L.

Abbreviations: BMI, body mass index; FT3, free 3,5,3′-triiodothyronine; FT4, free thyroxine; TPO, thyroid peroxidase; TRAb, thyroid receptor antibodies; TSH, thyrotropin.

### Primary Outcome

The number of participants in remission 2 years after a single dose of RTX and a 12-month course of ATD was 13, and because this exceeded the critical number of 9, the null hypothesis that the remission rate is less than 20% was rejected. The proportion of participants in remission was 0.48 (90% one-sided CI, 0.345-1).

Four participants attended their 2-year visit outside the allowable ± 14 day visit window; however, 3 of them had already relapsed by that time (receiving ATD after 12 months), and the remaining participant remained in remission at the next visit after the scheduled window.

Of the 13 participants in remission at 24 months, 2 had a raised TRAb antibody titer and a low serum TSH suggesting that there was a high likelihood of relapse beyond the 2-year trial window (Table 3). Of the 14 participants who relapsed, 13 received ATD between 12 and 24 months, and the remaining patient returned low TSH and high FT3 levels at 24 months. No patient received RI or surgical thyroidectomy.

**Table 3.** Summary of patient biochemistry and remission status

Patient ID	ATD dose at 1 y, mg (CBZ/PTU)	ATD in second y	Thyroid function and TRAb 2 y post-RTX					
			TSH	TSH low	FT3	FT3 high	TRAb	Remission
1	15	Yes	**67.34**	**No**	**2.9**	**No**	Pos	No
**2**	5	**No**	**0.06**	**Yes**	**5.5**	**No**	Neg	**Yes**
**3**	50	**No**	**1.13**	**No**	**6.0**	**No**	Neg	**Yes**
4	5	Yes	**6.76**	**No**	**4.8**	**No**	Neg	No
**5**	No ATD	**No**	**0.16**	**Yes**	**5.0**	**No**	Neg	**Yes**
6	2.5	Yes	**3.65**	**No**	**4.5**	**No**	Pos	No
**7**	No ATD	**No**	**0.08**	**Yes**	**6.5**	**No**	Pos	**Yes**
**8**	5	**No**	**0.56**	**No**	**5.0**	**No**	Neg	**Yes**
9	5	No	**0.05**	**Yes**	**19.3**	**Yes**	Pos	No
10	10	Yes	**0.05**	**Yes**	**11.2**	**Yes**	NP	No
11	5	Yes	**0.26**	**Yes**	**4.8**	**No**	Pos	No
12	2.5	Yes	**0.05**	**Yes**	**33.0**	**Yes**	ND	No
13	2.5	Yes	**0.05**	**Yes**	**14.8**	**Yes**	Pos	No
**14**	2.5	**No**	**0.35**	**No**	**5.7**	**No**	Neg	**Yes**
15	5	Yes	**0.05**	**Yes**	**6.4**	**No**	Pos	No
16	No ATD	Yes	**2.6**	**No**	**5.1**	**No**	NP	No
**17**	10	**No**	**0.05**	**Yes**	7.5	**No**	Neg	**Yes**
18	60	Yes	**0.05**	**Yes**	**25.5**	**Yes**	Pos	No
**19**	5	**No**	**0.59**	**No**	**5.3**	**No**	Neg	**Yes**
**20**	2.5	**No**	**5.62**	**No**	**4.6**	**No**	Neg	**Yes**
21	5	Yes	**0.05**	**Yes**	**9.0**	**Yes**	Neg	No
**22**	5	**No**	**1.27**	**No**	**4.6**	**No**	Neg	**Yes**
23	27.5	Yes	**0.05**	**Yes**	**6.3**	**No**	Neg	No
24	5	Yes	**0.05**	**Yes**	**5.7**	**No**	Neg	No
**25**	10	**No**	**1.53**	**No**	**5.6**	**No**	Neg	**Yes**
**26**	5	**No**	**0.81**	**No**	**5.5**	**No**	Neg	**Yes**
**27**	5	**No**	**0.05**	**Yes**	**6.5**	**No**	**Pos**	**Yes**

Biochemical relapse requires a TSH of less than 0.3 mU/L and serum FT3 greater than 7.7 pmol/L (age < 18 years) or 6.8 pmol/L (age > 18 years).

TRAb positive greater than 1.8 U/L; TRAb negative less than 1.8 U/L.

Abbreviations: ATD, antithyroid drug; CBZ, carbimazole; FT3, free 3,5,3′-triiodothyronine; FT4, free thyroxine; ID, identification; ND, not determined; Neg, negative; NP, not performed; Pos, positive; PTU, propylthiouracil; RTX, rituximab; TRAb, thyroid receptor antibodies; TSH, thyrotropin.

### Secondary Outcomes

#### Thyroid receptor antibody titers at rituximab administration

**Figure 3.**
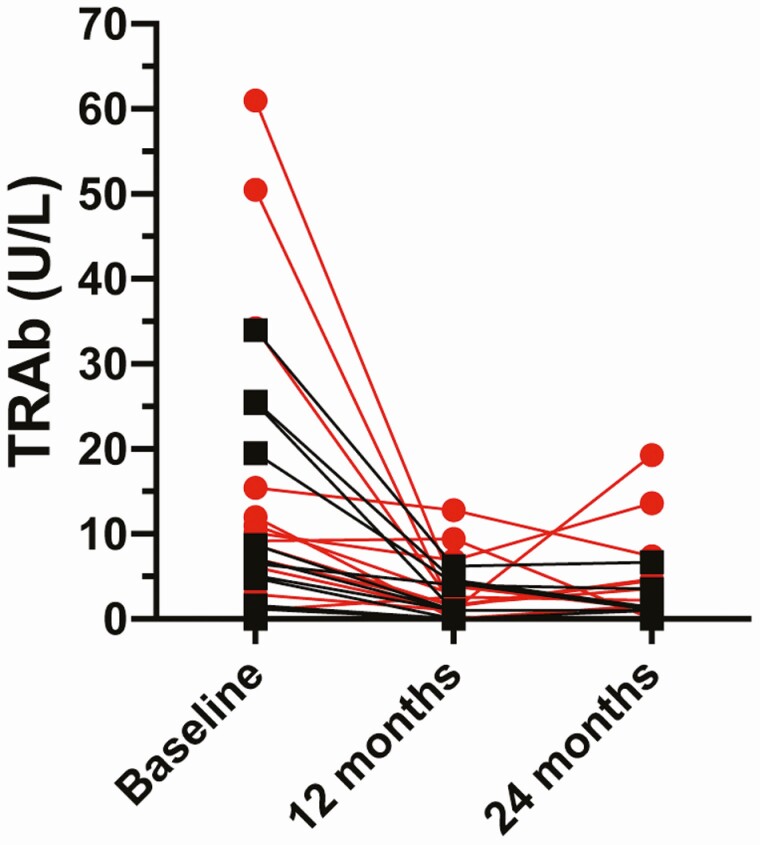
Thyroid receptor antibody (TRAb) titers measured centrally at baseline, 12 months, and 24 months. Patients who relapsed are shown in red.

TRAb titers, measured centrally, fell between baseline and the end of year 1 (Fig. 3). The median baseline TRAb titer in the remission group (measured centrally) was 6.50 U/L compared to 9.65 U/L in the relapse group. There was no evidence of a difference in median TRAb titers between the groups: difference in medians 3.15 (95% CI, –7.24 to 13.54; *P* = .30).

#### B-cell lymphocyte count

**Figure 4.**
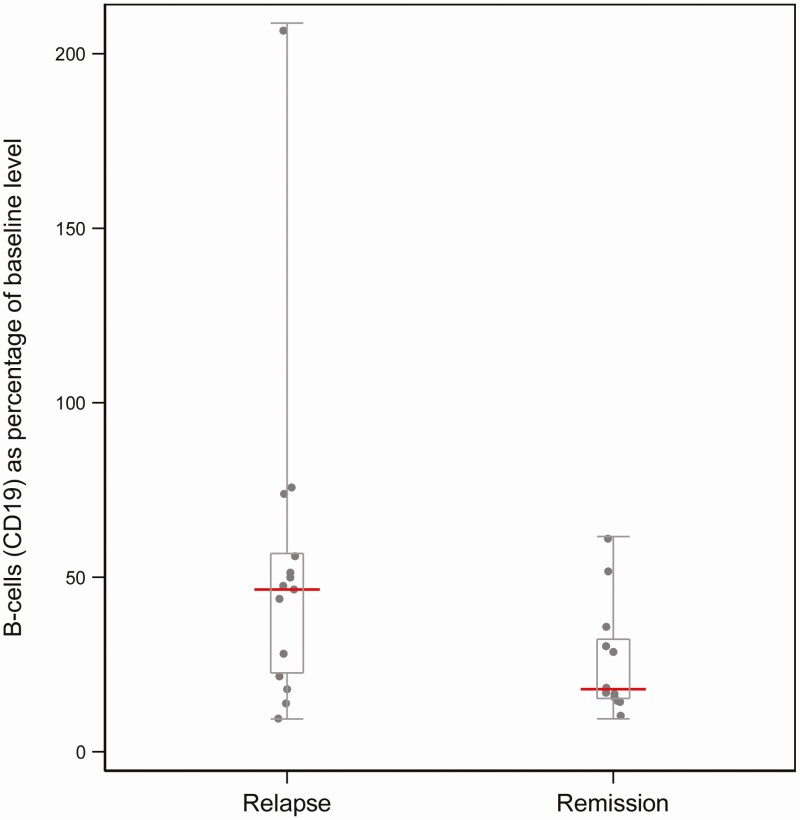
Box plot showing the distribution of B-cell lymphocyte count, expressed as a percentage of baseline value, at 28 weeks (visit 7) compared between patients in and out of remission: the red line indicates the median.

The median B-cell lymphocyte count compared to baseline at 28 weeks, expressed as a percentage of peripheral blood lymphocytes, was 18.0 in the remission group compared to 46.5 in the relapse group (Fig. 4). There was evidence of a difference between the groups in the median B-cell count: difference in median 28.5 (95% CI, 8.14-48.9; *P* = .064).

The median B-cell percentages at 52 weeks in the remission and relapse groups were 48.9 and 62.8, respectively, with no evidence of a difference (difference = 13.9, 95% CI, –9.0 to 36.9, *P* = .33).

### Antithyroid Drug (ATD) Dose at 12 Months and Cumulative ATD Dose

A total of 26 patients were commenced on CBZ and one 17-year-old patient was commenced on PTU. In the following description, 1 mg of CBZ is deemed to be equivalent to 10 mg of PTU in this patient. The median CBZ dose at the end of the first 12 months was 5 mg (range, 0-60 mg). Of the 27 participants, 24 were on a dose of 10 mg CBZ or less and 21 were on 5 mg or less, including 3 participants in whom the CBZ had been stopped (see Table 3). There was no evidence of a difference in the cumulative dose of CBZ between participants in remission, 54.6 mg/kg, and relapse, 60.5 mg/kg groups. The difference in median cumulative dose of CBZ (mg/kg) was 5.81 (95% CI, –48.7 to 60.4, *P* = .41).

### Time Taken for Thyrotropin to Rise

There was no clear evidence of a difference in the time taken for relief of TSH suppression between participants destined to achieve sustained remission (46 days) and in those who would relapse (93 days). The difference in median days for relief of TSH suppression was 47, (95% CI, –14.3 to 108.3; *P* = .17).

### Time Taken for Free 3,5,3′-Triiodothyronine and Free Thyroxine to Normalize

There was no clear evidence of a difference in the time taken for FT3 to normalize (return to within the normal age-related reference range) between participants who would subsequently achieve sustained remission (median, 27 days) and in those who would relapse (median, 58 days). The difference in median days for FT3 to normalize was 31 days (95% CI, –34.4 to 96.4; *P* = .24).

There was no difference in the time taken for FT4 to normalize (return to the normal age-related reference range) between participants destined to remit (31 days) or relapse (44 days). The difference in median days for FT4 to normalize was 13 (95% CI, –48.2 to 74.2; *P* = .73).

### Safety and Tolerability

Trial medications were well tolerated by all participants with no patient needing to stop CBZ or PTU because of side effects. All of the prescribed doses of RTX were given with only the well-described minor infusion-related symptoms experienced by some individuals. All participants completed the trial.

There were 363 AEs reported, of which 6 were serious adverse events (SAEs); none of the SAEs were found to be causally related to the trial interventions (Table 4). Of the nonserious AEs 333 were mild and 24 moderate in severity. There were 13 adverse reactions (ARs) considered possibly related to treatment, 4 probably related, and a further AE was not assessable (patient reported dizziness). Only 1 of the 17 ARs was moderate in severity; the remainder were considered mild. The mean number of ARs per participant was 0.6 (range, 0-3). The worst grade AR reported was moderate for 1 participant and mild for 8 participants; 18 participants did not experience an AR. Details of nonserious AEs/ARs according to system organ class are shown in Table 5. ATD (which can cause neutropenia) and RTX (which can cause hypogammaglobulinemia) had the potential to alter immune function and associated susceptibility to infections but no serious infections were noted. A summary of infections is shown in Table 6. No patient developed agranulocytosis or hypogammaglobulinemia. There was no discernible change in pathogen-specific antibody titers with no patients developing low pneumococcal or Hib antibodies during the trial and no increase in the number of patients with relatively low tetanus antibody titers (4 [out of 27] at baseline and 1 patient [out of 21 in whom levels were measured] at visit 14). ATPO were elevated at baseline in 20 patients out of 25 (not measured in 2). At 1 year ATPO titers had fallen in 18 out of 19 of these 20 participants (not measured at 1 year in 1).

**Table 4.** Severe adverse reactions experienced by trial participants

Patient ID	Description	MedDRA preferred term	Severity	Relationship to treatment	Seriousness criteria	Action taken in relation to SAE	Outcome
A	Admitted to hospital with abdominal pain	Abdominal pain	Severe	Unrelated	Hospitalization	Con Med, hospital	Recovered
B	Right thumb abscess with right forearm cellulitis	Abscess limb	Severe	Unrelated	Hospitalization	Hospital	Recovered
C	Noncardiac chest pain and bradycardia	Chest pain	Moderate	Unrelated	Hospitalization	Hospital	Recovered
D	Episodes of altered consciousness. Possible epilepsy	Nervous system disorder	Moderate	Unlikely	Hospitalization	Hospital	Recovered with sequelae
E	Epileptic seizure	Epilepsy	Moderate	Unrelated	Hospitalization	Hospital	Recovered
F	Hyperglycemia secondary to steroids	Hyperglycemia	Severe	Unrelated	Hospitalization	Nondrug therapy, hospital	Recovered with sequelae

Abbreviations: Con Med, Concomitant Medication; ID, identification; MedDRA, Medical Dictionary for Regulatory Activities; SAE, severe adverse event.

**Table 5.** Number and percentage of all participants (N = 27) affected by each nonserious adverse event or adverse reaction, reported by system organ class

System organ class	Total		Mild		Moderate	
	No.	%	No.	%	No.	%
Nervous system disorders	22	81.5	22	81.5	0	0.0
Respiratory, thoracic and mediastinal disorders	22	81.5	22	81.5	0	0.0
Infections and infestations	21	77.8	21	77.8	0	0.0
General disorders and administration site conditions	15	55.6	15	55.6	0	0.0
Gastrointestinal disorders	14	51.9	14	51.9	0	0.0
Musculoskeletal and connective tissue disorders	14	51.9	14	51.9	0	0.0
Psychiatric disorders	12	44.4	12	44.4	0	0.0
Injury, poisoning, and procedural complications	9	33.3	7	25.9	2	7.4
Reproductive system and breast disorders	9	33.3	8	29.6	1	3.7
Cardiac disorders	8	29.6	7	25.9	1	3.7
Eye disorders	8	29.6	8	29.6	0	0.0
Immune system disorders	8	29.6	7	25.9	1	3.7
Skin and subcutaneous tissue disorders	8	29.6	7	25.9	1	3.7
Investigations	6	22.2	6	22.2	0	0.0
Endocrine disorders	4	14.8	4	14.8	0	0.0
Surgical and medical procedures	3	11.1	2	7.4	1	3.7
Blood and lymphatic system disorders	2	7.4	2	7.4	0	0.0
Neoplasms benign, malignant, and unspecified (including cysts and polyps)	1	3.7	1	3.7	0	0.0
Metabolism and nutrition disorders	1	3.7	1	3.7	0	0.0
Vascular disorders	1	3.7	1	3.7	0	0.0

**Table 6.** Number of participants affected by infections and infestations according to system organ class term

Event term: infections and infestations	Total		Mild		Moderate	
	No.	%	No.	%	No.	%
Nasopharyngitis	15	55.6	15	55.6	0	0
Viral infection	7	25.9	7	25.9	0	0
Tonsillitis	4	14.8	4	14.8	0	0
Urinary tract infection	4	14.8	3	11.1	1	3.7
Upper respiratory tract infection	3	11.1	3	11.1	0	0
Coxsackie viral infection	1	3.7	1	3.7	0	0
Influenza	1	3.7	1	3.7	0	0
Lower respiratory tract infection	1	3.7	1	3.7	0	0
Nail infection	1	3.7	1	3.7	0	0
Otitis media	1	3.7	1	3.7	0	0
Parvovirus infection	1	3.7	1	3.7	0	0
Pharyngitis	1	3.7	1	3.7	0	0

## Discussion

This trial has indicated that a single dose of RTX may alter the clinical course of Graves hyperthyroidism. In the absence of RTX, we predicted a remission rate of between 20% and 30% after a 12-month course of ATD, which is generous given that such rates are usually seen after a course of 1 to 3 years’ duration (1-3). What we observed in this trial was a remission rate that was closer to 50% when a 12-month course of ATD was combined with a single baseline dose of RTX. It might be argued that a further 2 of the remission participants were at high risk of relapse but the remission rate would still have been around 40%, and there were additional pointers toward an effect of RTX on disease course. RTX targets the mature B cell and there was a relationship between B-cell number and outcome, with a lower percentage baseline B-cell count at week 28 associated with disease remission. This suggests a biological link between the action of the intervention and disease outcome although the difference in percentage B-cell count at week 28 did not reach a point where it could be used to predict outcome reliably. We also noted a remarkably low ATD drug dose around the time that ATD stopped at 12 months. Twenty-one of the 27 participants were on 5 mg CBZ or equivalent, or less.

There is increasing interest in exploring the potential role of novel immunomodulatory agents in autoimmune endocrine diseases in adults (21). The immunomodulatory impact of RTX has been documented in a number of different diseases including adults with GD and Graves orbitopathy (13-17). Many studies of RTX in adults have involved the administration of at least 2 doses, and our selection of one single dose was in part linked to a study in patients with Graves orbitopathy (16). We adopted this trial design because we did not believe there was sufficient evidence to proceed directly to a randomized trial in young people that would have involved a much larger number of participants and that would have been much more costly as a result. We have seen an efficacy signal in this clinical trial and feel that the next step is to conduct a randomized, concurrently controlled trial of a combination of RTX and CBZ in young people with Graves disease. Such a trial has not been conducted in young people or adults to date.

Young people were willing to take part in the trial, with most individuals approached agreeing to take part. We did not feel that the trial schedule was particularly onerous, with 1-day case admission for RTX infusion as the major additional intervention—the overall visit schedule was similar to that used in routine clinical practice in UK institutions. We were encouraged by the fact that all participants finished the trial with no SAEs linked to the treatment regimen. One patient developed what was suspected to be a seizure disorder during the course of the trial but he was subsequently diagnosed with pseudoseizures.

We were keen to focus on the potential effect of the treatment regimen on immune function, and there were no serious infections and no long-term hypogammaglobulinemia in this trial. There was no evidence of an effect on specific antibodies (tetanus/pneumococcus/Hib). RTX has been linked to neutropenia as an off-target effect (22) but we did not see any evidence of this in this trial. Nonetheless, there is no a priori reason to believe that patients with GD would be protected from uncommon or rare AEs associated with RTX immunotherapy in other contexts, and that the present trial was not powered to detect. We did note a reduction in ATPO titer in the majority of participants during the first year of the trial, which is likely to reflect the immunomodulatory action of RTX and/or ATD.

Although there was no evidence of any difference between the patients who remitted and relapsed in the time taken for thyroid function tests to normalize, the absolute number of days taken was smaller in the remission group for TSH, FT3, and FT4 concentrations. Longer-term remission rather than return to a euthyroid state is a more clinically relevant primary outcome that would be realistic in a larger trial.

A key question raised by this clinical trial is whether RTX is simply delaying the return of GD. We noted that when hyperthyroidism returned in relapse participants it was frequently profound from a clinical and biochemical perspective, suggesting a “rebound” effect. One potential driver may be a secondary rise in the concentration of B-cell activating factor (BAFF), already increased in the context of autoimmune disease, in response to RTX (23). Whether patient response in part reflects the BAFF/BAFF-binding receptor interaction and response post-RTX requires exploration, although in the context of patients with rheumatoid arthritis the picture is likely more complicated (24).

The single dose of RTX that we used in this clinical trial reflected earlier studies in patients with thyroid autoimmunity, although there is scope for the treatment regimen to be refined depending on factors such as the relationship between remission and immune response, which may be revealed in future studies. Patients with more severe GD are less likely to respond to thionamide ATD but whether this also applies to RTX cannot be settled without greater experience of its clinical use.

To summarize, this trial has indicated that RTX may alter the clinical course of Graves hyperthyroidism in the young, with no unacceptable side effects, so a formal randomized controlled trial is warranted.

## Data Availability

Some or all data sets generated during and/or analyzed during the present study are not publicly available but are available from the corresponding author on reasonable request.

## Abbreviations

AE: adverse event
AR: adverse reaction
ATD: antithyroid drug
ATPO: thyroid peroxidase antibodies
BAFF: B-cell activating factor
CBZ: carbimazole
FT3: free 3,5,3′-triiodothyronine
FT4: free thyroxine
GD: Graves disease
Hib: *Haemophilus influenza* group B
Ig: immunoglobulin
MedDRA: Medical Dictionary for Regulatory Activities
PTU: propylthiouracil
RI: radioiodine
RTX: rituximab
SAE: serious adverse event
T4: thyroxine
TRAb: thyroid receptor antibodies
TSH: thyrotropin

## Additional Information

***Disclosures:*** S.H.S.P. discloses consulting for Apitope and speaker fees from Merck, Sanofi, Berlin Chemie, and Quidel. T.D.C. discloses speaker fees from Novo Nordisk and Pfizer. The other authors have nothing to disclose.
